# Exploring adults’ motives for food choice of sustainable diet components: a qualitative study in Tehran Metropolis

**DOI:** 10.1186/s40795-021-00459-7

**Published:** 2021-10-01

**Authors:** Arezoo Haghighian Roudsari, Abouali Vedadhir, Samira Pourmoradian, Hania Rahimi-Ardabili, Maryam Shokouhi, Ali Milani-Bonab

**Affiliations:** 1 Department of Community Nutrition, Faculty of Nutrition Sciences and Food Technology, National Nutrition and Food Technology Research Institute, Shahid Beheshti University of Medical Sciences, PO Box: 19395-4741, No. 7, Hafezi, Farahzadi Blvd., Shahrak Qods, Tehran, 1981619573 Iran; 2 Department of Anthropology, Faculty of Social Sciences, University of Tehran, Tehran, Iran; 3 Population Health Sciences, University of Bristol, Canynge Hall, Bristol, UK; 4 Nutrition Research center, Tabriz University of Medical Sciences, Tabriz, Iran; 5 Centre for Primary Health Care and Equity, University of New South Wales, Sydney, NSW Australia; 6 Nutrition Research Australia, Sydney, NSW Australia; 7 Department of Food and Nutrition Policy and Planning, Faculty of Nutrition Sciences and Food Technology, National Nutrition and Food Technology Research Institute, Shahid Beheshti University of Medical Sciences, Tehran, Iran

**Keywords:** Sustainable diet, Food choice, Qualitative study

## Abstract

**Background:**

Todays, due to the impact of human food choices on increasing greenhouse gas emissions, water consumption and environmental degradation, there is a new approach about changing the pattern of food production and consumption, including sustainable food and nutrition system related to consumption. This study aimed to explore the components of a sustainable diet among the factors that affect people’s food choices.

**Methods:**

This qualitative study was carried out using an in-depth interview with 33 individuals aged 30–64 years old living in different areas of Tehran. Data collection, data analysis and theoretical conceptualization were performed simultaneously. MAXQDA 10 software was used for managing and organizing the data.

**Results:**

In this paper, the findings are categorized according to the key components of a sustainable diet in five themes: “Health and Nutrition”, “Food and Agriculture Security”, “Environment and Ecosystems”, “Markets, food trade and production chains”, “social, cultural, and policy factors” were categorized. Meanwhile, the components of the “Health and Nutrition” domain had the highest contribution and the components of the two domains “food and agriculture” and “environment and ecosystems” had the lowest role based on the participants’ perception in this study.

**Conclusion:**

Considering to the low importance of the components of a sustainable diet in food choices of the community, promoting the individual awareness of sustainable diet components, clarifying the importance of food choices in creating environmental impacts and leading the national macro policies in the field food and nutrition toward sustainable diet goals are essential.

**Supplementary Information:**

The online version contains supplementary material available at 10.1186/s40795-021-00459-7.

## Background

Nowadays, worldwide demographic, epidemiologic, and nutrition transitions have led to an increase in the prevalence of obesity and non-communicable diseases, such as, Type 2 diabetes and cardiovascular diseases [1]. Despite the sluggish economic growth, the nutritional transition in Iran has occurred in the context of urbanization, rapid demographic and social capital changes, so we face with another concept of malnutrition [2]. In the past few years, malnutrition was associated with protein and energy deficiencies, and today it is transformed into abdominal satiety and cellular hunger due to lack of micronutrients in the current diet. While the malnutrition and micronutrient deficiency still persists, obesity and related issues have emerged seriously, especially between women in urban areas [3].

The worrying speed of climate and environmental change, and its destructive effects on food, nutrition and health systems, new thinking has currently emerged about changing the pattern of food production and consumption that includes sustainable food systems and nutrition. There has been a rising tendency in the food and diets sustainability which we consume today and in the future [4, 5]. In the field of food production and consumption, sustainability has been dramatically impacted by extensive environmental implications of agriculture and meat production, which include land use, water resources, greenhouse gas emissions and waste production [6, 7]. The term sustainable diet was first introduced in 1986 by Gussow and Clancy (25) A sustainable healthy diet is known as a diet that, preserves biodiversity and ecosystems; promotes individuals’ health; and is culturally accepted, accessible, safe and economically fair [8].

It is necessary to determine the components of sustainable diet from environmental, biological, cultural and health perspectives, at the worldwide, regional, local and individual levels [9]. From the sustainable nutrition approach, the diet is not simply about the production and consumption of human food. What consumers buy and consume will affect not only their health but also their environment [10]. The meals we eat have different effects on greenhouse gas emissions due to their protein and calorie content [11, 12]. Globalization is related to changes in people’s daily food choices which have potentially negative impacts on the environment [13]. Urbanization and increased incomes are accompanied by increased consumption of diets high in meat, dairy, oil, salt, and processed foods [14]. At the same time, the globalization of the food system has contributed to environmental degradation and biodiversity loss [15]. In addition, recent evidence indicated that the global food system is responsible for 30% of global greenhouse gas emissions (GHGEs) and climate change as a result of rising demand to transport, store, and consume the most resource-intensive food types (namely dairy and meat) in developing economies will further increase the contributions of food and agriculture to environmental degradation and consumers have an enormous role to reduce the production of these gases by modifying their consumption of animal foods [9, 16, 17]. Thus, it can be understood that the position of food choices is very important in the pursuit of sustainable development goals. Big Knowing people’s perceptions of sustainable diet concepts and their impact on food choices has the potential to plan and implement sustainable nutrition interventions and policies. A recent systematic review that aimed to identify the main drivers towards adopting sustainable dietary behaviors indicated that attitude and intention are the most recurrent significant behavioral predictors [18]. Previous studies show that individuals might have limited knowledge about the effects of their food choice on the environment [14, 15]. For example, the results of a recent qualitative study which was assessed Australian food shopper perceptions, experiences and attitudes towards healthy and environmentally friendly foods, showed that compared to health, the relationship between food and the environment is rarely considered by consumers [19]. Similarly, in another work, consumers have also been reported to have less awareness and perceptions about the association between food that they consumed and climate changes [20]. Given that various factors such as cultural, educational and environmental factors might affect individuals’ perception on the sustainable diet, it is crucial to examine it in the context of the Iranian population to inform future interventions and policies on sustainable food choices in Iran and countries with similar contexts. Therefore, we explored individuals’ perception on the sustainable diet, motivations for following/unfollowing a sustainable diet, and facilitators and barriers to consuming a sustainable diet.

## Methods

### Study framework

We analyzed participants response based on a framework developed by Downs et al. [21]. This framework categorizes the key components of a sustainable diet within five domains. The first domain of “Nutrition and Health” covers the components related to the health and disease-related to nutrition such as obesity, and type 2 diabetes. The second domain is about the importance of sustainable diet on “Food Security and Agriculture” such as nutritional quality of food produced and water use and the third domain of “Environment & Ecosystems” is composed of components on the management of energy and resources as well all pesticide uses. The “Markets, Trade and Value Chains for Economic Growth”, the fourth domain, contains economic determinants such as food availability/affordability (e.g. food price); and food import and export. Finally, the “Sociocultural and Political factors” encompasses components like animal welfare, food equity, food knowledge and skill.

### Study design and participants

This constant comparative analysis study using qualitative design to deeply explore adults’ perception of food choice determinants which related to sustainable nutrition components. We used RATs guideline to report the study [22].

A purposive sampling strategy was used to recruit participants from different residency areas in Tehran (capital of Iran) during early 2019. Online means such as emails and physical posters on the university campus were used to recruit participants. Participant inclusion criteria were: aged 30–64 years, considering the maximum variety in socio-economic characteristics such as education, type of job. In addition, participants were invited to take part in the research after explaining the study objectives and selected based on their availability and willingness to participate and the ability to communicate their verbally.

After interviewing 29 participants, the answers were repetitive and we reached data saturation, meaning that no new findings were found from participants’ interviews. However, four other people were interviewed to ensure that the data was not repetitive and Sampling was completed with 33 participants.

### Data collection, management and analysis

One-on-one, in-person semi-structured interviews were conducted to explore the determinants of food choice from the perspective of the participants. First, the participants were explained about the objectives of the research, and after obtaining consent, a demographic questionnaire including marital status, education level, household type and dimension, residence areas, and ethnicity was completed. An interview protocol was developed by AHR and AV to guide the interviews including questions about participants’ opinion regarding five domains of sustainable diet including “Nutrition and Health”, “Food Security and Agriculture”, “Environment & Ecosystems”, “Markets, Trade and Value Chains for Economic Growth”, and the “Sociocultural and Political factors” and also. The main body of interview protocol included the following open-ended questions: Could you please describe the foods and meals you may eat during the day? What are the factors that influence your food choice? What do you think about traditional and indigenous food? What are the foods you choose in different seasons of the year? What is your choice of food in different places and times such as holidays, parties? Participants were encouraged to talk about their thoughts regarding to these questions.

The interview was piloted by role-playing of research team members with one researcher being interviewer and the other as interviewee to ensure that it would generate constructive data. Prior to the interview, participants filled a demographic questionnaire on gender, age, occupation,

Interviews lasted 30 to 45 min and were conducted by the chief researcher (AHR) in a private place. The interviews were audio recorded using digital dictation voice recorders (Olympus DS-2500) and transcribed verbatim by a professional transcription service. Data collection and analysis and theoretical conceptualization were carried out concurrently from the beginning of the research to ensure theoretical saturation. Interview data were analyzed thematically [23], mainly using a deductive approach.

The process of data analysis was performed applying open, axial, and selective coding stages proposed by Strauss & Corbin [24]. The software MAXQDA 10 was used to manage and organize the data. In the next step, the components of the sustainable diet were sought and analyzed from the perspective of individuals to determine whether participants’ views were consistent with the components of a sustainable diet.

Participants’ key statements were identified, open codes similar in meaning were classified into subcategories. In axial coding, based on the proximity of their purports, subcategories were grouped into larger categories. Main themes emerged by integrating the categories with due attention to their properties.

### Reflexivity, data validity and trustworthiness

To ensure the validity of data, researchers spent enough time on interviewing, coding, and reviewing the concepts. Participants were provided with an opportunity to review and check whether the transcripts accurately reflected what they said (i.e. respondent validation); none of the participants expressed any concern. Important field notes, nonverbal reactions of the participants, and memos were recorded during the interviews.

Participants were also selected on the way to maximize diversity in the area of residence, occupation, and education to ensure data credibility and reproducibility. The researchers attempted to abandon their previous assumptions so as not to interfere with the process of interviewing, conceptualizing, and interpreting the data to achieve conformability. Interviews were initially coded by the first author [AHR], the interviewer and researchers were all Iranian and familiar with the cultural background. The interviews, open and axial codes to ensure reliability were reviewed by the all research team. Transferability of data was done by clarifying the participants characteristic, data collecting methods, interpreting and conceptualizing the data and reporting some quotations which the participants expressed.

## Results

In this study, the participants’ statements are classified according to the key components of sustainable diet in five themes: “Health and Nutrition”, “Food and Agriculture Security”, “Environment and Ecosystems”, “Markets, Food trade and production chains”, “Social, Cultural, and Policy factors”.

Table 1 represents the demographic characteristics of the participants in the qualitative study. The conceptualization of participants’ statements showed that among the determinants of food choice in adults, there were some concepts which classified for a sustainable diet (Table 2).

- Domain 1: Health and Nutrition

**Table 1:** Participants’ characteristics in this study (*n* = 33)

Demographic traits	Frequency (%)
Gender	
Female	22 (66.66)
Male	11 (33.33)
Age (years)	
30–49	21 (64)
50–64	12 (36)
Education	
Primary	10 (30)
Diploma	6 (18)
University	17 (52)
Marital status	
Married	30 (91)
Single	3 (9)
Employment status	
Employed	20 (61)
Housekeeper	13 (39)

**Table 2:** Explored sustainable nutrition components from among food choice determinants in the study participant (*n* = 33)

Sustainable nutrition domains	Explored sustainable nutrition components
**Health and Nutrition**	Food diversity, diet quality
	Exercise, physical activity and/or sedentary lifestyle
	Food safety and sanitation
	Malnutrition (overweight and obesity)
	Burden of non-communicable diseases, chronic disease, diet-related diseases
	Consumption of calories, macronutrients, processed foods, fat, sugar, and junk foods
**Food and Agriculture Security**	Seasonal foods, traditional and local foods
**Environment and Ecosystems**	Use of pesticides, herbicide, fertilizers
**Markets, food trade and production chains**	Proper infrastructure and access to markets, distance to market, transportation costs to market, storage
	Food availability and cost-effectiveness, food price, food environment
	Food marketing, advertisement, food packaging
**social, cultural, and policy factors**	Nutrition awareness
	Consumers’ compliance, taste, convenience, preferences
	Nutrition knowledge, cookery skills and training, food preparation,

Participants’ food choices in the domain of “Health and Nutrition” included several components of a sustainable diet. Observing food diversity involves the consumption of different food groups and avoiding repetitive food choices. Majority of participants stated that they try to choose the food which has better quality and this quality could be of high-quality raw materials, the appearance of foods, less use of preservatives, food hygiene and standardization:

*“I care about the quality of the food and I take care that the quality of the food does not go down. Usually, the food we prepare is standard food and my wife is very careful when cooking it to get high quality.” (Male, 62 years old)*

Physical activity level was one of the items that some participants mentioned and noted its importance in the amount and type of food consumed:

*“Since most days I go to sports or clubs or yoga, my breakfast is just a little bit of date, tea and walnut. I do not eat too much bread and cheese. I prefer to exercise with a light stomach and so I feel better. I mean, for me, dates and walnuts are good for breakfast.” (Female, 39 years old).*

According to the study participants, some of the foods they choose are influenced by their concerns about being overweight and losing their current fitness. Sometimes people would choose foods that they believed could improve their appearance by refraining from some foods such as sweets that might be harmful to them.

*“I like confectionary but try to eat less. Why? Because of its fat content. But my family eats. I eat sometimes” (Male, 62 years old)*

Non-communicable diseases such as diabetes were also a concern for choosing some foods for most of the participants. These sensitivities were increased particularly in the case of high-calorie foods containing sugar and fat.

*“When trying to buy meat, I try to get beef that has less fat, or when trying to buy yogurt or dairy, I try to get the low-fat variety. Well, anyway, obesity has many dangers.” (Male, 38 years old)*

*“I try to do not eat fat and salt in our food anymore. We don't eat at all, and my weight was too high. My weight was 106kg, I'm 80kg now, and I'm very happy. I find it much easier to live this way.” (Male, 63 years old)*

- Domain 2: Food and Agriculture Security

In the domain of “Food and Agriculture Security”, the majority of participants’ food choice centered on traditional, indigenous and local products. Many participants explained that because of the healthiness and usefulness of traditional foods, they preferred these foods to industrial food, but there were still those who had to choose foods containing the additive and the occasional preparation of traditional foods because of a busy lifestyle. The concept of indigenous, local and traditional foods was classified into two groups of having a healthy look and ethnic pattern of food.

*Having healthy look:* most of the Participants believed that the local food has a positive impact on health and wellbeing of themselves and their children compared to the industrial food that was considered unhealthy.

The positive impact of local food on the health and wellbeing of children, traditional food preference to industry due to unhealthy, healthy beliefs and the character of local food were statements expressed by majority of the interviewees about the local and indigenous foods.

Local and traditional foods are one of the most important cultural and indigenous symbols. Almost all of the foods that are cooked in one place and geographical area originate in the customs of that area. According to the most of the participants’ opinions in this study, local foods are better and healthier than modern foods and fast foods and have a very good effect on the health and disease status of the community:

*“We still eat some local foods sometimes. I taught my kids to eat these foods, for example, KASHK (an Iranian cuisine that derived from dairy products) have a lot of calcium. I think they were more proper because we had more power when we ate that kind of food. I'm healthier.” (Female, 39 years old)*

*“I think local food is much more effective. When my kids eat local food, I think they are much healthier and much better in terms of health” (Female, 43 years old)*

Despite the fact that majority of participants preferred traditional food, the time and skills required to prepare them, and the availability of ingredients prevent them from cooking these foods:

*“To cook “Abgoosht” [mutton soup thickened with chickpeas], you have to be in the kitchen from morning to noon, or to cook the “Ghorme Sabzi” (an Iranian stew which is a mixture of sautéed herbs, kidney bean and meat), and you have to wash the vegetables the night before, chop, fry then cook.” (Male, 62 years old)*

“I like traditional foods like beetroot “Ash [heavy soup]”. Or I use Ghee myself to cook “Halva” but my children don’t like it. But I do and I love it” (Female, 34 years old).

*Ethnic patterns of food:* Some of the participants talked about ethnic patterns of food, such as: preparing special traditional foods at a special occasion, reception, and ethnic training about the specific way of cooking, and cooking local foods when all family members have been gathered. Several participants believe special dishes are also served for many special occasions and ethnic patterns:

*“Sometimes in a special occasion that my family and others join, such as my mother-in-law, father-in-law, grandmother, grandfather or sisters, particular food such as local foods is prepared.” (Male, 62 years old).*

- Domain 3: Environment and ecosystems

Within the domain of “Environment and ecosystems”, the key components of the use of pesticides, herbicides and fertilizer were discussed among participants. Majority of Participants’ tendency to consume organic food was seen as one of the indicators of healthy food. Their knowledge of how to produce and process organic food made them able to choose organic food. Since organic food are produced with minimal use of chemical fertilizers, pesticides and hormones, they were a desirable option to consume for those people who were concerned about the contamination of food with toxins and hormones. However, in this study, there are some barriers to the consumption of organic foods, such as high cost and low availability according to the participants’ quotes:

*“These green [organic labeled] chickens are a bit more expensive than regular chickens. I haven't eaten so far. There are also ostrich and turkey meats that I rarely buy as I can’t afford them.” (Male, 43 years old)*

*“When spear thistle [a wild local plant] comes to market in spring and early summer, we eat a lot because we love it. We're consuming things that's natural more, for example, spear thistle grows in the mountains naturally, and has no fertilizer” (Male, 62 years old).*

*“I try to make our food organic so that we don't have to worry about it [pestsides and chemicals] anymore and feel comfortable eating it.” (Male, 63 years old).*

- Domain 4: Markets, food trade and production chains

In the domain of “Markets, food trade and production chains” and their role in the sustainable diet, most of the participants talked about several components. In this study, several participants pointed out components such as proper infrastructure and access to markets, distance to market, transportation costs to market, storage, food availability and food prices, food marketing in terms of advertising and food packaging:

*“I only care about my time, and whether I have enough space to store a food item in the fridge before I buy, or need it now. Some foods like some vegetables and fruit need preparations before storage, like carrots, if I want to buy them, I have to prepare them before I store. ”(Female, 36 years old)*

*“Ever since we have entered the mechanical and industrialized life system and with including new appliances such as fridge and freezer, our food has become more stored and frozen.” (Female, 61 years old)*

Economic feasibility was expressed as the most important factor in the food selection process. From the majority of participants’ point of view, many factors contributed to their food choices, but what ultimately played the most important role was the financial affordability of the food that could affect the quantity and quality of the food in various forms. In many cases, people were aware of the benefits of healthy foods, but due to economic constraints, it was not possible to supply of such foods:

*“As usual, family income is very important, on whether the foods that are healthy can be purchased” (Female, 36 years old).*

*“Sometimes we had no money, and we had to put aside meat and poultry and replace with the other foods such as textured soy protein and mushroom” (Female, 39 years old).*

According to some participants, they were exposed to widespread advertising by manufacturers, exporters, importers and via Internet networks every day. The majority of those believed that foods promoted through the press and mass media, especially radio and television, were safe, healthy and harmless. According to the study participants, media advertising played a significant role in their understanding of the food and familiarity with reputable brands:

*“There are various messages delivered via advertisements, for example, one of them says “do not eat tilapia fish, it is harmful”. This message unconsciously affects me in a way that I may not eat fish anymore”. (Female, 39 years old).*

*“The educational messages we received from broadcasting or the media is very effective in changing people's lifestyles in some ways”. (Female, 36 years old).*

According to the most of interviewee, the type and appearance of the food packaging were one of the factors influencing their choices. This was especially evident when people went to grocery stores to shop. The good color of the packaging could be so effective that people were encouraged to choose that food because the packaging was attractive, particularly even when they had no previous plans for that food:

*“The food packaging is very important to me. When I go to Hypermarket, and I see the fruit items that are in packages which are very neat and clean, I would like to buy them”. (Female, 50 years old)*

*“In my choice of food, the packaging also has some influence. I like to be stylish”. (Female, 33 years old)*

- Domain 5: social, cultural, and policy factors

In the “social, cultural and political” domain of sustainable diet, some of the components such as nutrition awareness, consumer acceptance and taste preferences, knowledge, skills, education and nutritional literacy are mentioned in this study. People’s awareness of food properties, cooking skills, the effect of foods on health and disease influenced their food choices. Participants also pointed to the role of nutritional awareness in the quality of their food choices. According to them, nutritional knowledge was acquired through training classes, related books, newspapers and television programs and information gained through employment:

*“With this information and knowledge we get from people around or we hear in the media, or we read and acquire in the training class, our awareness has increased here, and we have learned how to make healthier foods”. (Female, 62 years old)*

Color, odor, and taste of food were some of the factors that the participants paid particular attention to when purchasing food, and even in some cases, they were considered as indicative of nutrients and healthiness of food. Many participants considered tastes as one of their main reasons for choosing food.

*“Taste is very important. I choose more sour foods than sweet ones”. (Female, 62 years old)*

The intrinsic tendency of the flavors, tastes and some of the special foods resulted in choosing food only according to taste and ignoring other criteria.

*“I consider more my interest and if I am not restricted, my taste is the maim drive for my choice”. (Female, 35 years old)*

Poor cooking skills were another reason why people were forced to abandon certain foods:

*“I like fish and seafood but one reason [for not consuming these foods is that] it's hard to cook at home, so I use them less”. (Female, 39 years old).*

## Discussion

The findings of the present study showed the what majority of participants considered important in choosing their food items includes only a small part of the key domains identified in the field of the sustainable diet. The “Health and Nutrition” domain was the main contributor. This result aligns with a recent systematic review’s findings highlighting the adoption of a healthy or low-fat diet as participants main intention in choosing food [18]. The components of two latter domains, including food production and sustainable agriculture; food aid; social support networks; subsidies and fiscal policies; soil and water pollution; and greenhouse gas emissions are generally impacted at the macro-level of policy-making scale. Thus, community dwellers may not be directly involved in the development of these domains.

Production and trade-related policies include national-level policies and interventions which govern what food is available, reasonably priced, and accessible for the community. These interventions should address a broad population at various levels such as individual, community and national; society groups (school, workplaces, food production Stakeholders) or food setting (grocery store and canteen); and sub-group people which categorized by different traits (socio-economic status, gender and age) [25].

Given the nature of the domains of Health and Nutrition, individual performance plays an increasingly important role in the social, cultural, and political areas. The safety of the food, which was mentioned by the participants was not specific to any particular social class, and people with any socio-economic status were concerned with the health and safety aspects of food. Most people expressed concerns about the contamination of food at the market. In other words, one of their concerns was the attempt to choose and prepare food that had the least contamination with possible chemicals and hormones. This finding is consistent with the Brimblecombe study, which examined Australian adults’ views about healthy food choice [26].

The environment and Ecosystems are the other domain that the majority of participants were less sensitive about them when choosing their food. According to all dietary guidelines, people must consume a variety of different foods to meet their nutrients needs. However, to reduce the environmental impact of food choices, it is necessary to decrease the consumption of certain foods such as red meat and dairy products. This change in food choice should be in a way that nutritional adequacy of diet is ensured. For instance, consumers should be aware of how to substitute red meat with low-fat plant foods, such as legumes [27]. Accordingly, aiming to put dietary patterns in line with the sustainability targets, governments should start to reconsider their national dietary guidelines in a way that ensure the public health, while protecting the environment in a sustainable manner [28] A recent study by Donati et al. found that achieving a sustainable diet is feasible by integrating environmental and economic sustainability, and this should be carefully considered by policymakers to reach multiple targets [29].

“Social, cultural, and policy factors” are another domain of sustainable diet which majority of our studied participants had considering to its components when choosing foods. Urban life characteristics, such as the lack of access to some food items, made it impossible for the participants of the study to prepare the traditional foods. Moreover, some other considerations, such as particular limitations which exist in the urban residences, do not allow participants to choose and cook some food items. Traffic and concerns about air and environmental pollution, as well as water and food contamination, may also influence people’s food choices and, sometimes force them to eliminate or substitute some food items. In another study, other barriers to climate-friendly food choices, including high food prices, convenience, and lack of awareness were identified [30]. Living in different geographical areas of the country forms a dietary pattern for individuals that is specific to the foods and variety of local agricultural products due to its climatic and ecological features. This result is also inconsistent with the Devine study of food choices [31]. Concerning food prices coming from “markets, food trade and production chains” domain, the cost of purchasing and preparing food has been an important factor in many studies regarding the food choice [26, 32, 33]. Since food prices are relevant issues as regarding sustainable diet affordability [34], policy makers should carefully consider the effectiveness and feasibility of fiscal measures, such as taxes or subsidies, in promoting healthier food choices [35].

Most of the participants considered tastes as one of their reasons for choosing food. The innate tendency of the flavors and the tastes may lead them to choose some food items, even by ignoring other criteria. In Blake’s study, people also tended to choose foods that fit their interests, and they also focused on their dietary and taste preferences [36].

Changes in behaviors that affect consumption appear to be an effective way of influencing dietary choices based on environmental conditions. One possible solution is to increase access to environment-friendly food at food supply centers such as restaurants and grocery stores to give consumers better options for food choices [37, 38]. Several interventions have been suggested to inspire healthy food choices, such as changing environments to make better the choices [39]. Nudging is the one of the main mechanism suggested by Thaler & Sunstein [40] which define as any aspect of the choice set that could predictably modify the people’s behavior without any changing in their economics incentives including alteration of physical components in environments, namely, awareness and necessary information.

The present study, similar to previous research showed that literacy plays a critical role in the quality and quantity of food selection. In other words, ethical and environmental-based food choices are an active, conscious and smart process that happens with sufficient awareness [41, 42]. Media and advertising are considered as the main source of information about food and nutrition for people [43]. In addition, the media has the capacity to convince people about some particular food choice. Studies have shown that both positive and negative advertising messages are associated with the formation of short-term changes in nutritional knowledge and attitudes, and even a nutritional message may have a direct effect on behavioral changes [44]. A series of the evidence also shows that women may reflect the information they receive about the sustainable diet in their food choice more than men do, and show significantly greater concern about the environmental sustainability when deciding about the food [45–47].

The finding showed that the “health and nutrition” dimension is over-perceived by the respondents is also in line with the findings of Biasini et al. (2021) showing that most of the studies published on the sustainable diet were focusing on the health/nutrition dimension and with this regard, an increase in consumers’ awareness seems needed [18].

## Conclusion

The findings of the present study showed that what participants considered as important in choosing their food items includes only a small part of the key domains identified in the field of the sustainable diet. To achieve the goals of sustainable nutrition, it is necessary to raise the awareness of people about the various aspects of a sustainable diet to be one of the most important strategies to be considered in Iranian society. In addition, the role of macro policymakers in planning and formulating effective policies to achieve sustainable dietary goals is of particular importance.

### Supplementary Information

**Additional file 1.**

## Data Availability

The datasets used and/or analysed during the current study are available from the corresponding author on responsible request.
