# Correction: Exploring adults’ motives for food choice of sustainable diet components: a qualitative study in Tehran Metropolis

**DOI:** 10.1186/s40795-023-00790-1

**Published:** 2023-11-27

**Authors:** Arezoo Haghighian Roudsari, Abouali Vedadhir, Samira Pourmoradian, Hania Rahimi-Ardabili, Maryam Shokouhi, Ali Milani-Bonab

**Affiliations:** 1 Department of Community Nutrition, Faculty of Nutrition Sciences and Food Technology, National Nutrition and Food Technology Research Institute, Shahid Beheshti University of Medical Sciences, No. 7, Hafezi, Farahzadi Blvd., Shahrak Qods, PO Box: 19395-4741, Tehran, 1981619573 Iran; 2 Department of Anthropology, Faculty of Social Sciences, University of Tehran, Tehran, Iran; 3 Population Health Sciences, University of Bristol, Canynge Hall, Bristol, UK; 4 Nutrition Research center, Tabriz University of Medical Sciences, Tabriz, Iran; 5 Centre for Primary Health Care and Equity, University of New South Wales, Sydney, NSW Australia; 6 Nutrition Research Australia, Sydney, NSW Australia; 7 Department of Food and Nutrition Policy and Planning, Faculty of Nutrition Sciences and Food Technology, National Nutrition and Food Technology Research Institute, Shahid Beheshti University of Medical Sciences, Tehran, Iran

**BMC Nutr 7, 55 (2021)**.

10.1186/s40795-021-00459-7.

Following publication of the original article [1], the authors reported a significant number of corrections that were missed in the original publication. The changes do not affect the article’s results or conclusions. The key changes to note are the following:

**On the page 3;** line 13; in the left column: the year “early 2019” should be corrected as “early 2015”.

**On the page 3**; In the sub-heading “Data collection, management and analysis”: The text of this sub-heading is modified as follows:

**Corrected paragraph**: The data used in this study is related to a qualitative study entitled “Explaining and examining of food choice process model using mixed method among adults aged 30–64 living in Tehran” with ethical ID: IR.SBMU.nnftri.Rec.1393.053556, which was conducted in early 2015. An interview protocol was developed by AHR and AV to guide the interviews. The main body of interview protocol included the following open-ended questions: Could you please describe the foods and meals you may eat during the day? What are the factors that influence your food choice? What do you think about traditional and indigenous food? What are the foods you choose in different seasons of the year? What is your choice of food in different places and times such as holidays, parties?

**On the page 3;** line 16–18; in the right column: This sentence was added to the end of the line 18: “based on the domains of Downs et al’ framework.”

**Corrected sentence**: “Interview data were analyzed thematically [23], mainly using a deductive approach based on the domains of Downs et al’ framework.”
