# Supplementary material for: Exploring adults’ motives for food choice of sustainable diet components: a qualitative study in Tehran Metropolis
Source: BMC Nutr. 2021 Oct 1;7:55. doi: 10.1186/s40795-021-00459-7 (PMC8485460; doi:10.1186/s40795-021-00459-7)
Supplement: Supplementary file 1 — Additional file 1. [file 40795_2021_459_MOESM1_ESM.docx]

**Supplementary file**

This questionnaire was prepared with the aim of **"Examining the factors affecting food choice among adults living in Tehran (Capital of Iran)".** Your detailed answers to the questions in this questionnaire will help us identify and prioritize key factors in food selection, followed by citizen health planning. It should be noted that each person's answers are confidential. Thank you very much for working with us.

**General information**

- **Gender:** Female € Male€
- **Age:** ……. (Yrs)
- **Occupational status:** Housekeeper € Retrieved € University student € Unemployed € Having occupy €
- **Education level:** Illiterate € Elementary literacy € Diploma € University education €
- **Martal status:** Single € Married €

**Semi-structured Interview Guide**

This interview lasts about 45 minutes, which is about your experiences with food choices, and there are no right or wrong answers, and you cannot answer some questions. If you are satisfied, the interview will be recorded with the device. The purpose of this study is to increase our understanding of the food choice in adults living in Tehran.

- Could you please describe the foods and meals you may eat during the day?
- What are the factors that influence your food choice?
- What do you think about traditional and indigenous food?
- What are the foods you choose in different seasons of the year?
- What do you think is the role of environmental factors in our food choices?
- What is your choice of food in different places and times such as holidays, parties?
